# Gastrointestinal Basidiobolomycosis Mimicking Crohn's Disease in a Young Adult

**DOI:** 10.7759/cureus.100395

**Published:** 2025-12-30

**Authors:** Ahmer A Longi, Misbah Fazlani, Maida Balila, Yevginiy Karamurzin, George Alexander

**Affiliations:** 1 Internal Medicine, Mediclinic Welcare Hospital, Dubai, ARE; 2 Infectious Disease, Mediclinic Welcare Hospital, Dubai, ARE; 3 Pathology, Mediclinic Welcare Hospital, Dubai, ARE; 4 Gastroenterology and Hepatology, Mediclinic Welcare Hospital, Dubai, ARE

**Keywords:** antifungals, colonoscopy, gastrointestinal basidiobolomycosis, infectious colitis, inflammatory bowel disease

## Abstract

Gastrointestinal basidiobolomycosis (GIB) is a rare, emerging fungal infection caused by *Basidiobolus ranarum*. It primarily affects immunocompetent individuals and often presents as a mass lesion mimicking colorectal malignancy or inflammatory bowel disease. Due to its non-specific presentation and rarity, GIB is frequently misdiagnosed, leading to delayed or inappropriate management. In this article, we present a case of GIB in a young male in his 20s who presented with a four-month history of intermittent and recurrent severe abdominal pain associated with significant weight loss. Based on the information at hand, he was being treated for a suspected inflammatory bowel disease. Subsequently, he underwent a gastroscopy and colonoscopy. His colonoscopy initially reported him to have acute severe destructive colitis with a wide differential diagnosis, including inflammatory bowel disease, Bechet’s disease, ischemic or pseudomembranous colitis. Based on initial suspicion, the patient was started on therapy for Crohn's disease, with which he showed mild improvement. However, he again got worse with exacerbating symptoms. His histopathology was sent to a tertiary care hospital in the United States, and he was found to have GIB. After initiation of antifungal therapy, including a combination of itraconazole and intravenous liposomal amphotericin (later changed to voriconazole), his symptoms showed remarkable improvement. This case report highlights the importance of recognizing the rare presentation of GIB in an immunocompetent adult who was initially misdiagnosed as a case of inflammatory bowel disease, emphasizing the importance of histopathological evaluation in diagnosing fungal infections. It also indicated that GIB, despite being a highly destructive colitis, can be successfully treated with antifungals without the need for invasive surgical procedures.

## Introduction

Gastrointestinal basidiobolomycosis (GIB) is an uncommon yet increasingly reported fungal infection caused by *Basidiobolus ranarum*, a saprophytic fungus typically found in soil, decomposing organic matter, and the gastrointestinal tracts of amphibians and reptiles [1]. Originally recognized as a causative agent of subcutaneous infections, *B. ranarum* has more recently been implicated in visceral mycoses, particularly affecting the gastrointestinal tract. The majority of reported cases have emerged from tropical and subtropical regions, notably Saudi Arabia, Iran, and certain areas of the United States [2]. The reservoir of basidiobolomycosis includes soil and vegetation along with the gastrointestinal tract of amphibians and reptiles. GIB is thought to be acquired by ingestion of infected material. The clinical findings will include gastrointestinal symptoms with bloody diarrhea, abdominal pain, and weight loss. Imaging studies may reveal focal or multifocal bowel wall thickening, colonic masses, or hepatic abscesses, which can mimic conditions such as inflammatory bowel disease, diverticulitis, or intra-abdominal malignancies [3]. Histopathological features, such as the Splendore-Hoeppli phenomenon (fungal organism surrounded by eosinophilic infiltrate), are essential for accurate diagnosis and effective treatment.

Due to the absence of distinctive clinical or radiological features, a high index of suspicion is essential, with definitive diagnosis relying on histopathological confirmation [4]. Despite being documented in fewer than 150 cases, GIB has become an important differential diagnosis in patients presenting with abdominal pain, gastrointestinal masses, or nonspecific systemic symptoms such as fever and weight loss [5]. GIB frequently mimics malignancies or inflammatory bowel diseases, complicating early and accurate diagnosis [6]. We present the case of a young male who presented with gastrointestinal symptoms mimicking inflammatory bowel disease or tuberculosis. Through investigations, leading to a biopsy of the gastrointestinal tract was able to led us to a diagnosis of a rare gastrointestinal fungal infection.

## Case presentation

A male student in his 20s, known to have attention-deficit hyperactivity disorder (ADHD) and currently studying in a foreign country, presented to the emergency department with a four-month history of recurrent severe abdominal pain, primarily localized to the central and right abdominal regions. He reported an unintentional weight loss of approximately 20 kilograms during this period. While in another country, he had been hospitalized twice for similar symptoms, where a presumptive diagnosis of subacute intestinal obstruction was made. He was found to have a positive fecal occult blood test, but tests for *Clostridium difficile* were negative.

Initial imaging in another country revealed gross thickening of the cecum and ascending colon, with associated inflammatory changes, including pericolic stranding, lymphadenopathy, and pneumatosis intestinalis. There was no evidence of perforation or abscess formation. Upper gastrointestinal endoscopy showed non-specific gastritis.

**Figure 1:**
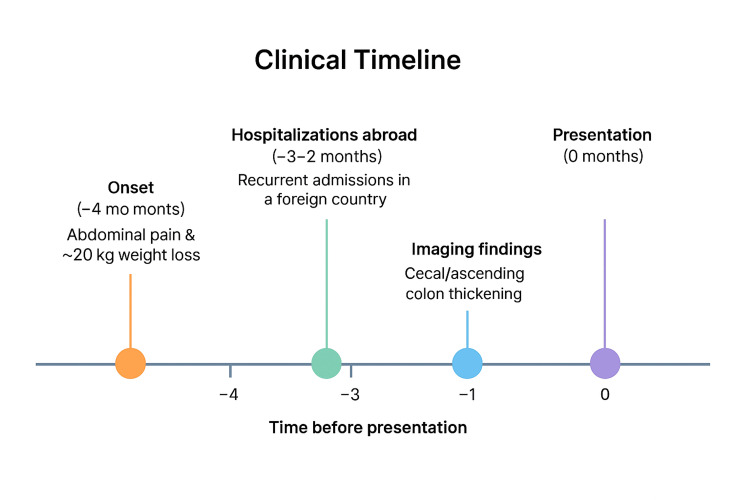
Clinical timeline of the patient’s disease course.

Upon arrival, his vital signs included blood pressure 138/82 mmHg, heart rate 114 bpm, temperature 36.6°C, respiratory rate 17 breaths/min, and oxygen saturation 98% on room air. Physical examination revealed localized tenderness in the right upper quadrant and right iliac fossa without guarding or rebound tenderness. There were no signs of jaundice, clubbing, lymphadenopathy, or peripheral stigmata of chronic disease. Cardiopulmonary examinations were unremarkable (Figure 1).

Laboratory investigations (Table 1) revealed leucocytosis, neutrophilia, eosinophilia, anemia (Hb 11.5 g/dL), thrombocytosis, hypoalbuminemia, and elevated C-reactive protein. Faecal calprotectin was markedly elevated at 501.6 μg/g. Liver and renal function tests were within normal limits. Serologic testing for viral hepatitis, HIV, and tuberculosis (via Quantiferon-TB Gold) was negative. The stool analysis did not show any presence of parasites.

**Table 1:** Laboratory investigation CRP: C-reactive protein; ALT: alanine aminotransferase; AST: aspartate aminotransferase; INR: international normalized ratio; PTT: partial thromboplastin time; MRSA: methicillin-resistant *Staphylococcus aureus*; HBsAg: hepatitis B surface antigen; HCV: hepatitis C virus; PT: prothrombin time

Labs	Results	Reference ranges
Hemoglobin	11.5 g/dL	13.0-17.5 g/dL
Neutrophils	13.39 x 10^3^/μL	1.70-7.60 x 10^3^/μL
Lymphocytes	1.36 x 10^3^/μL	1-3.20 x 10^3^/μL
Eosinophils	1.36 x 10^3^/μL	0-0.5 x 10^3^/μL
Platelets	513 x 10^3^/μL	150-450 x 10^3^/μL
CRP	68.5 mg/L	2.15-2.5 mmol/L
Faecal calprotectin	501.6 ug/g	Negative:<50, indeterminate 50-120, positive >120 ug/g
Lipase	13.4 U/L	13-60 U/L
Total bilirubin	5.53 umol/L	<21.0 umol/L
Direct bilirubin	3.41 umol/L	<7.0 umol/L
Alkaline phosphate	88 U/L	40-129 U/L
ALT	16.7 U/L	<50 U/L
AST	16.3 U/L	<50 U/L
Albumin	27.3 g/L	35-52 g/L
Sodium	139 mmol/L	136-145 mmol/L
Potassium	4.0 mmol/L	3.50-5.10 mmol/L
Chloride	99.5 mmol/L	98-107 mmol/L
Bicarbonate	26.9 mmol/L	22-28 mmol/L
Calcium	2.49 mmol/L	2.15-2.5 mmol/L
Magnesium	0.70 mmol/L	0.66-1.07 mmol/L
Phosphate	1.08 mmol/L	0.81-1.45 mmol/L
PT	14.9 seconds	11.7-15.3 seconds
INR	1.02	0.80-1.20 ratio
PTT	38.8 seconds	28.6-40.0 seconds
MRSA	Negative	Negative
HBsAg	Non-reactive	Non-reactive
Anti-HCV	Non-reactive	Non-reactive
Quantiferon TB Gold Test	Negative	Negative

**Figure 2:**
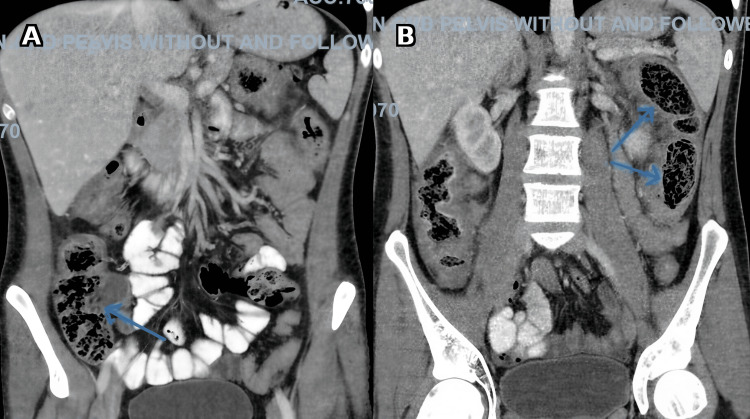
Contrast-enhanced CT of the abdomen Coronal images show luminal narrowing at the ascending colon (blue arrow) (A) and at the splenic flexure and descending colon (blue arrows) (B).

He was admitted and started on intravenous hydration along with intravenous steroids (hydrocortisone 100 mg three times daily). A contrast-enhanced CT scan of the abdomen and pelvis (Figure 2) demonstrated circumferential thickening and luminal narrowing of the colon, particularly the ascending and transverse colon, with submucosal fat deposition, perienteric stranding, mural enhancement, and significantly enlarged mesenteric and paracolic lymph nodes, radiologically suggestive of Crohn’s disease.

**Figure 3:**
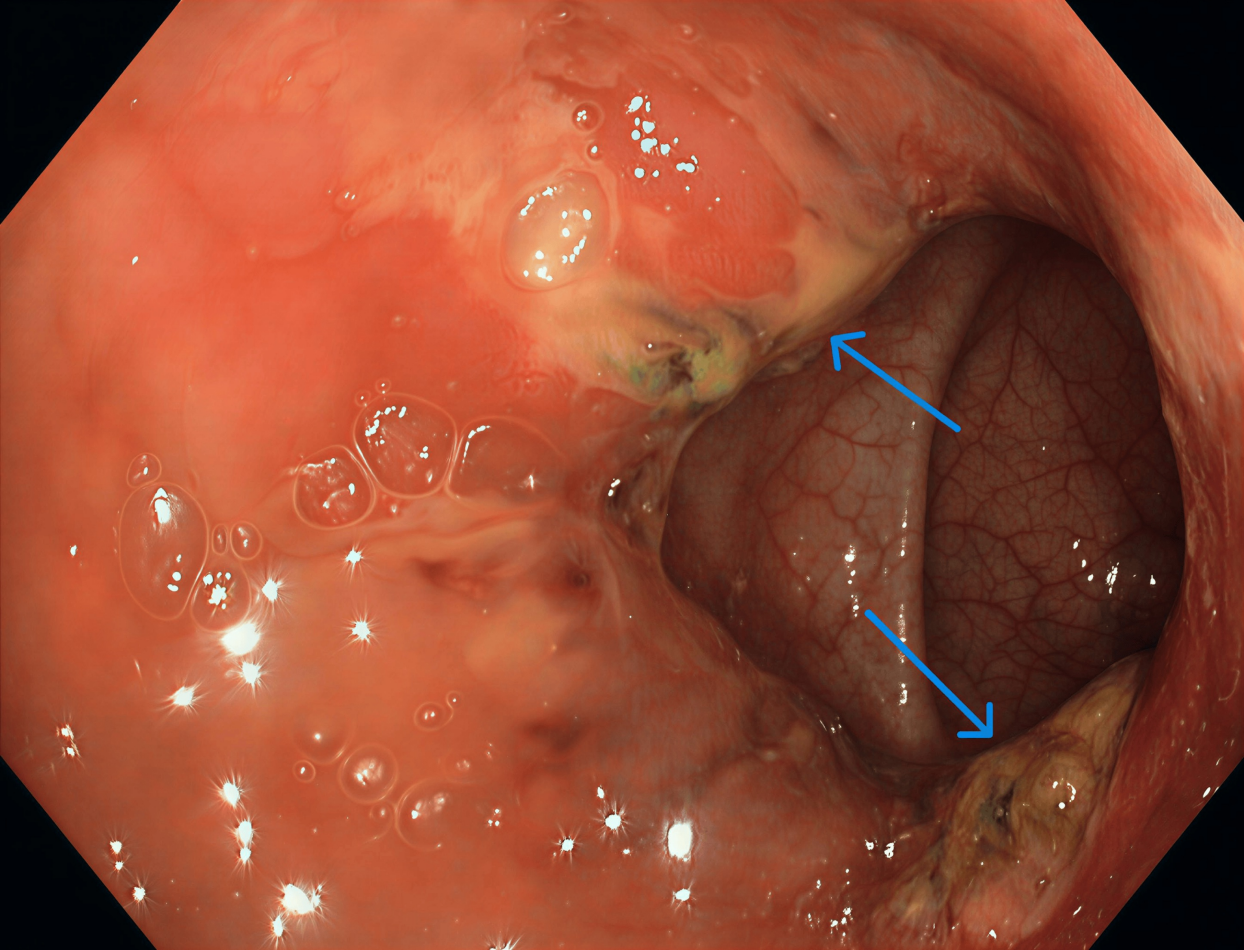
Colonoscopy findings showing multiple skip areas of linear ulcers with nodularity and erythema (blue arrows)

**Figure 4:**
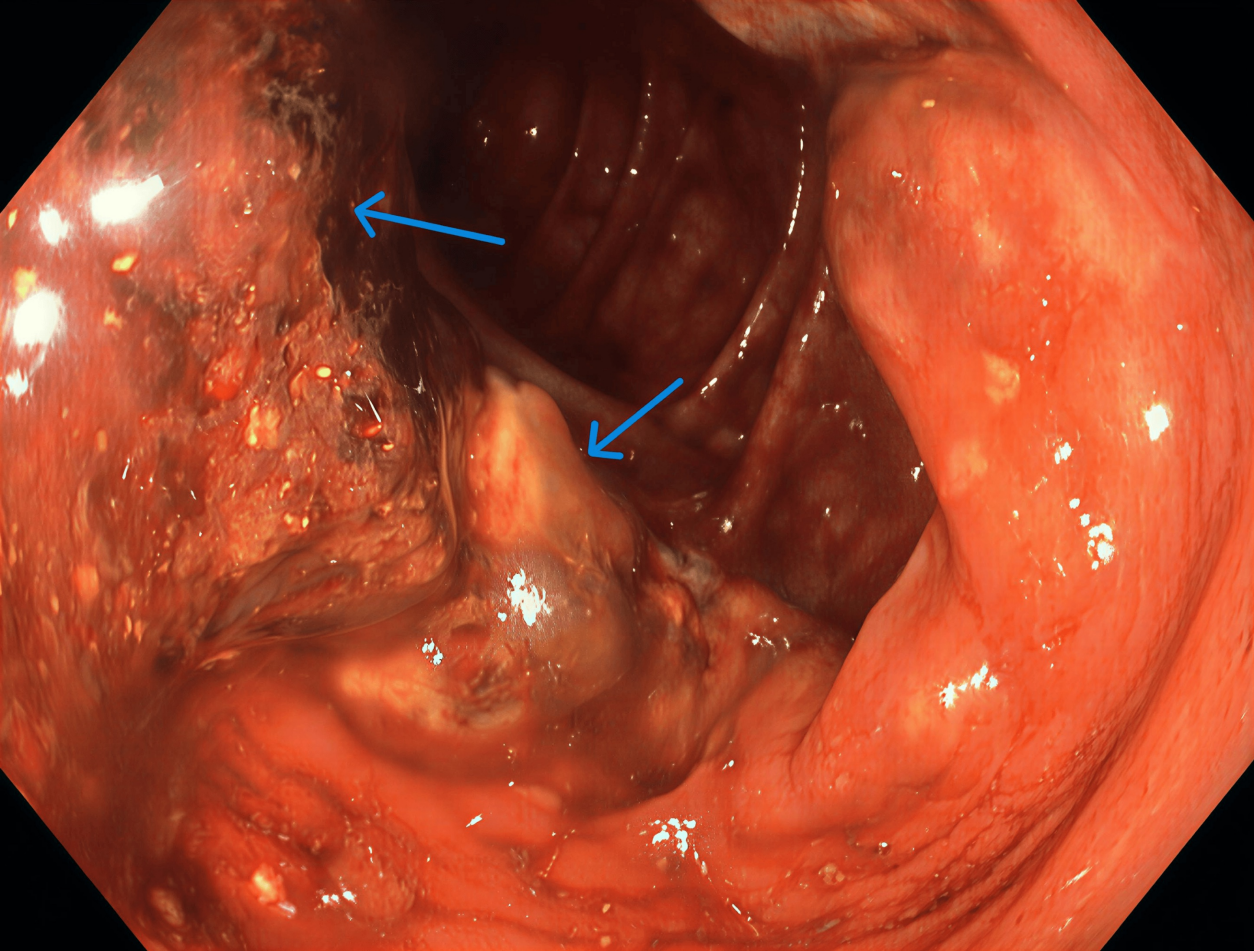
Colonoscopy findings showing destructive colitis with nodularity and erythema (blue arrows)

Subsequently, upper and lower gastrointestinal endoscopies were performed. Gastroscopy revealed multiple antral ulcers with sloughing, and biopsies showed reactive gastropathy. Colonoscopy (Figures 3-4) demonstrated multiple skip lesions, deep linear ulcers, nodularity, and luminal narrowing in the ascending, transverse, descending, and sigmoid colon, with normal intervening mucosa and terminal ileum. The mucosa appeared firm and scirrhous on biopsy.

Histopathological examination of the colonic biopsies showed severe diffuse acute destructive colitis in all sampled segments, with no evidence of granulomas, acid-fast bacilli, fungal organisms, parasites, or viral inclusions. Given the extensive inflammation, a differential diagnosis including inflammatory bowel disease, Behçet’s disease, ischemic colitis, and pseudomembranous colitis was considered. The duodenal biopsy was unremarkable.

The patient showed clinical improvement after initiation of intravenous hydrocortisone and was planned to be started on biological therapy with risankizumab (600 mg) for presumed Crohn’s disease. He tolerated oral intake, and his abdominal symptoms abated. He was discharged in stable condition. Meanwhile, the colonic biopsy samples were sent to Mayo Clinic for expert histopathological review for further diagnostic clarification.

**Figure 5:**
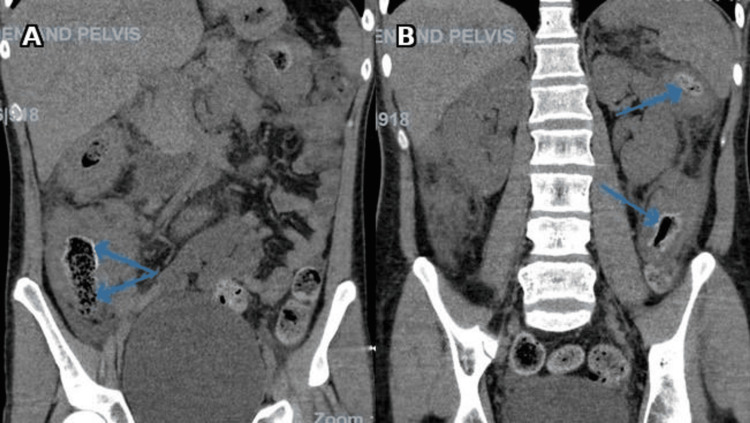
Contrast-enhanced CT of the abdomen and pelvis Coronal images show worsening luminal narrowing of the ascending colon with associated edema at the ileocecal junction (blue arrows) (A), and worsening luminal narrowing at the splenic flexure and descending colon (blue arrows) (B).

**Figure 6:**
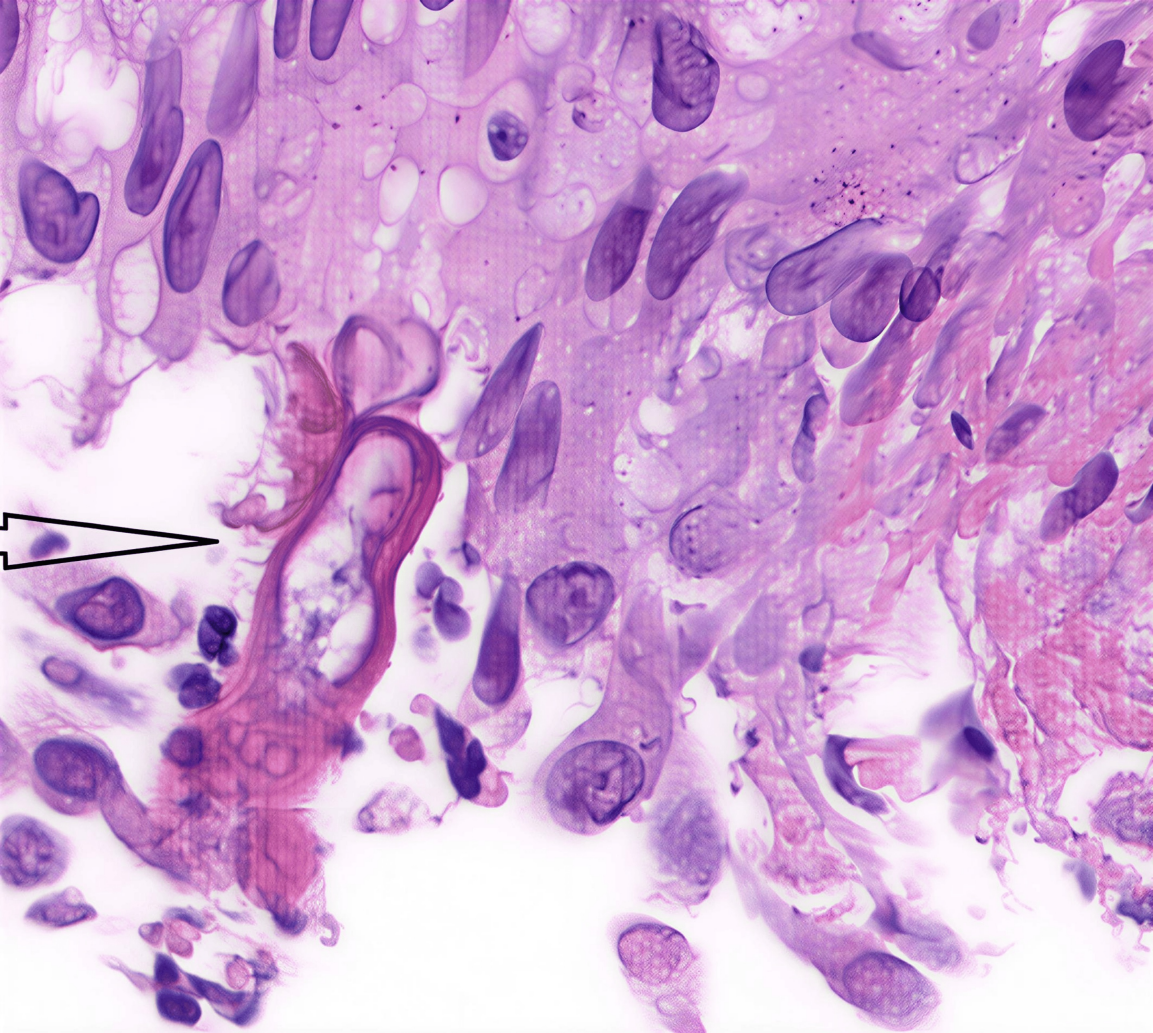
Histopathological findings Microscopic examination reveals a small cluster of epithelioid macrophages surrounding fungal hyphae and a zygospore of *Basidiobolus* species, highlighted by periodic acid-Schiff (PAS) with diastase stain (black arrowhead). Immunohistochemistry shows the epithelioid macrophages to be positive for CD163 and negative for S100, CD1a, Langerin, and CD25.

The patient, however, was admitted again with abdominal pain. Repeat imaging with CT abdomen (Figure 5) showed worsening of luminal narrowing of the ascending, transverse, and descending colon with an edematous wall. The histopathological review of his biopsies indicated a rare fungal infection consistent with basidiobolomycosis (Figure 6). Stains for cytomegalovirus (CMV) and tuberculosis were negative. Infectious disease was also consulted, and after a multidisciplinary team meeting, it was decided to start the patient on antifungal medications with a combination of itraconazole and liposomal amphotericin B (5 mg/kg once daily), which was eventually changed to voriconazole: loading dose 6 mg/kg twice daily for two doses, followed by oral dose 200 mg twice daily. This was in combination with itraconazole 200 mg once daily. The patient was discharged on voriconazole and itraconazole.

## Discussion

The clinical and radiological ambiguity surrounding GIB often results in misdiagnosis. In multiple reported cases, such as the one described by Rizk et al., imaging findings of necrotic masses and hepatic lesions led to an initial misdiagnosis of metastatic colorectal carcinoma [3]. Likewise, Meeralam et al. reported a case that was initially misdiagnosed as Crohn's disease, attributed to the presence of fistulizing lesions and a chronic inflammatory clinical course [2].

Histopathological analysis remains the definitive method for diagnosis. Hallmark features include granulomatous inflammation accompanied by a prominent eosinophilic infiltrate and fungal elements encased in eosinophilic material, a finding referred to as the Splendore-Hoeppli phenomenon [1,7]. In numerous instances, these diagnostic features are recognized only postoperatively, following extensive surgical resections performed under the initial assumption of malignancy [6,8]. Eosinophilia can be a valuable diagnostic clue in basidiobolomycosis (especially GIB), but it is not diagnostic by itself, and its absence does not rule out the disease. Tissue eosinophilia on biopsy, often with the Splendore-Hoeppli phenomenon (eosinophilic material around fungal elements), which is a classic pathologic pattern in GIB.

A major challenge in the management of GIB is the lack of standardized treatment guidelines, largely due to its rarity. Antifungal agents such as itraconazole or voriconazole are generally effective, especially when administered early in the course of the disease [3,5]. In advanced or complex cases, surgical resection may be required to address complications such as obstruction, perforation, or tissue necrosis [1,6]. Soleimani et al. described a case that was initially mistaken for ulcerative colitis but subsequently progressed to a disseminated infection necessitating multi-organ resection, with a definitive diagnosis of GIB established through histopathological examination [6].

Untreated GIB carries a mortality rate of up to 16%, with potential complications such as bowel perforation, peritonitis, and systemic dissemination [7]. In the case reported by Mobarki et al., the patient with GIB died from sepsis secondary to colonic perforation [9]. This emphasizes the importance of early detection and aggressive treatment.

A distinctive feature of this infection is its tendency to affect immunocompetent individuals, frequently in the absence of significant predisposing factors. This contrasts with most invasive fungal infections, which primarily occur in immunocompromised patients, thereby adding to the diagnostic challenge [1,10].

Although radiologic findings are non-specific, they commonly reveal bowel wall thickening, mesenteric masses, and hepatic lesions that can mimic metastatic malignancies or abscesses. Accurate diagnosis requires a comprehensive approach that integrates clinical presentation, imaging studies, and histopathological evaluation [3].

Due to the geographic concentration of cases in the Middle East and tropical regions, environmental exposure, likely through the ingestion of contaminated food or water, has been proposed as the primary mode of transmission [1,4]. However, the occurrence of numerous cases in non-endemic regions highlights the importance of maintaining global clinical awareness of this condition [8].

## Conclusions

GIB is a rare but serious fungal infection that poses significant diagnostic challenges due to its ability to mimic malignancy and inflammatory bowel conditions. Increased awareness among clinicians, especially in endemic regions or when faced with atypical gastrointestinal presentations, is essential. Prompt histopathological diagnosis and initiation of antifungal therapy, potentially combined with surgical intervention, are crucial for successful outcomes. Clinicians must maintain a high index of suspicion, especially when conventional diagnoses fail to account for the patient’s presentation or treatment response fully.

## References

[1] Mirmoosavi S, Salehi M, Fatahi R (2023). Gastrointestinal basidiobolomycosis - a rare fungal infection: challenging to diagnose yet treatable - case report and literature review. IDCases.

[2] Meeralam Y, Alsulami H, Aljoaid AM, Khayat M, Zahrani S, Khairo M, Alotaibi S (2023). Basidiobolomycosis mimicking fistulizing Crohn’s disease: a case report from Saudi Arabia. Cureus.

[3] Rizk RC, Yasrab M, Weisberg EM, Fishman EK (2024). Gastrointestinal basidiobolomycosis masquerading as cancer. Radiol Case Rep.

[4] Alsaeed M, Mursi M, Bahloul A, Alrumeh A, Arab N, Alrasheed M (2023). A rare case of fatal gastrointestinal basidiobolomycosis. IDCases.

[5] Alabdan L, Amer SM, Alnabi Z, Alhaddab N, Almustanyir S (2020). Gastrointestinal basidiobolomycosis in a 45-year-old woman. Cureus.

[6] Soleimani N, Anbardar MH, Nikoupour H (2024). Disseminated gastrointestinal basidiobolomycosis: a case report with review of diagnostic clues. Case Rep Med.

[7] Balkhair A, Al Wahaibi A, Al-Qadhi H, Al-Harthy A, Lakhtakia R, Rasool W, Ibrahim S (2019). Gastrointestinal basidiobolomycosis: beware of the great masquerade a case report. IDCases.

[8] Fathaddin A, Alobaid S, Alhumoudi D, Almarshoud G, Alsubaie A, Alotaibi NH (2024). Gastrointestinal basidiobolomycosis: a rare manifestation of Basidiobolus ranarum in a non-endemic region. J Surg Case Rep.

[9] Mobarki M, Alhakami N, Ahmad M (2024). Basidiobolomycosis: unusual cause of colonic perforation. Cureus.

[10] Alsharidah A, Mahli Y, Alshabyli N, Alsuhaibani M (2020). Invasive basidiobolomycosis presenting as retroperitoneal fibrosis: a case report. Int J Environ Res Public Health.

